# Developing and Validating a Self-Care Self-Efficacy Scale for Oral Anticoagulation Therapy in Patients With Nonvalvular Atrial Fibrillation: Protocol for a Mixed Methods Study

**DOI:** 10.2196/51489

**Published:** 2024-09-13

**Authors:** Arianna Magon, Jeroen Hendriks, Rosario Caruso

**Affiliations:** 1 Health Professions Research and Development Unit IRCCS Policlinico San Donato San Donato Milanese Italy; 2 Caring Futures Institute College of Nursing and Health Sciences Flinders University Adelaide Australia; 3 Centre for Heart Rhythm Disorders The University of Adelaide and the Royal Adelaide Hospital Adelaide Australia; 4 Clinical Research Service IRCCS Policlinico San Donato San Donato Milanese Italy; 5 Department of Biomedical Sciences for Health University of Milan Milan Italy

**Keywords:** oral anticoagulation therapy, nonvalvular atrial fibrillation, self-care, self-efficacy, medication adherence, patient-centered education, scale development, validation, psychometrics, atrial fibrillation, education, development, anticoagulation, prevention, stroke, medication, management

## Abstract

**Background:**

Oral anticoagulation therapy (OAC) is the cornerstone treatment for preventing venous thromboembolism and stroke in patients with nonvalvular atrial fibrillation (NVAF). Despite its significance, challenges in adherence and persistence to OAC regimens have been reported, leading to severe health complications. Central to addressing these challenges is the concept of self-efficacy (SE) in medication management. Currently, there is a noticeable gap in available tools specifically designed to measure SE in OAC self-care management, while such tools are crucial for enhancing patient adherence and overall treatment outcomes.

**Objective:**

This study aims to develop and validate a novel scale aimed to measure self-care self-efficacy (SCSE) in patients with NVAF under OAC, which is the patients’ Self-Care Self-Efficacy Index in Oral Anticoagulation Therapy Management (SCSE-OAC), for English- and Italian-speaking populations. We also seek to assess patients’ SE in managing their OAC treatment effectively and to explore the relationship between SE levels and sociodemographic and clinical variables.

**Methods:**

Using a multiphase, mixed methods observational study design, we first conceptualize the SCSE-OAC through literature reviews, patient focus groups, and expert consensus. The scale’s content validity will be evaluated through patient and expert reviews, while its construct validity is assessed using exploratory and confirmatory factor analyses, ensuring cross-cultural applicability. Criterion validity will be examined through correlations with clinical outcomes. Reliability will be tested via internal consistency and test-retest reliability measures. The study will involve adult outpatients with NVAF on OAC treatment for a minimum of 3 months, using both e-surveys and paper forms for data collection.

**Results:**

It is anticipated that the SCSE-OAC will emerge as a reliable and valid tool for measuring SE in OAC self-care management. It will enable identifying patients at risk of poor adherence due to low SE, facilitating targeted educational interventions. The scale’s validation in both English and Italian-speaking populations will underscore its applicability in diverse clinical settings, contributing significantly to personalized patient-centered care in anticoagulation management.

**Conclusions:**

The development and validation of the SCSE-OAC represent a significant advancement in the field of anticoagulation therapy. Validating the index in English- and Italian-speaking populations will enable personalized patient-centered educational interventions, ultimately improving OAC treatment outcomes. The SCSE-OAC’s focus on SCSE introduces a novel approach to identifying and addressing individual patient needs, promoting adherence, and ultimately improving health outcomes. Future endeavors will seek to extend the validation of the SCSE-OAC across diverse cultural and linguistic landscapes, broadening its applicability in global clinical and research settings. This scale-up effort is crucial for establishing a universal standard for measuring SCSE in OAC management, empowering clinicians and researchers worldwide to tailor effective and culturally sensitive interventions.

**Trial Registration:**

ClinicalTrials.gov NCT05820854; https://tinyurl.com/2mmypey7

**International Registered Report Identifier (IRRID):**

PRR1-10.2196/51489

## Introduction

### Overview

Oral anticoagulation therapy (OAC) represents the primary and secondary prevention choice for venous thromboembolism and stroke in patients with nonvalvular atrial fibrillation (NVAF) [1]. As a life-long treatment, OAC is prescribed for various clinical indications, such as heart failure, congenital heart disease, hematological disorders, and peripheral vascular disease, affecting approximately 2% of the Western population. Despite its efficacy, the quality of anticoagulation control remains a challenge, impacting patient safety, efficacy in preventing thromboembolic events, and overall well-being. With an aging population, OAC use is expected to increase due to the equally increasing prevalence of AF [1].

Research has highlighted patients’ low adherence and persistence to OAC, leading to preventable thromboembolic and hemorrhagic complications like stroke and bleeding. Addressing the underlying health determinants of patient adherence is essential to develop personalized patient-centered educational interventions [2]. Among these determinants, patients’ self-efficacy (SE) plays a crucial role in empowering patients to actively engage in self-care management. SE is associated with patient knowledge and motivation, enabling timely medication intake and self-care activities even under uncertain and challenging conditions [3-5], and will contribute to shared decision-making in OAC treatment [6].

Despite the extensive research on the OAC population, inconsistent results and underreporting of SE persist [3]. One of the main reasons for this inconsistency is the heterogeneity of tools used to measure SE in this specific context. Existing tools, such as the Chronic Disease Self-Efficacy (CDSES) [7], General Self-Efficacy (GSE) [8], and Self-Efficacy for Appropriate Medication Use Scale (SEAMS) [9], have a broad focus and are not tailored to assess the specific SE aspects relevant to OAC self-care management. Consequently, there is a need for a dedicated tool that can capture these challenges and optimize OAC treatment outcomes. In other words, the effects of SE measured using broad tools on specific OAC outcomes might be misleading because the available general tools are not fit to measure aspects of SE that might impact anticoagulation control safety and other OAC-specific results in anticoagulation therapy management, as stated in a recent study [10].

### Self-Care Self-Efficacy Framework

A self-care self-efficacy (SCSE) framework emerges as a valuable theoretical model for developing a measure of OAC-specific SE by being rooted in the middle-range theory of self-care for chronic illness [11,12] and Bandura’s [13] social cognitive theory. In this framework, the SCSE is the patient’s confidence in self-care maintenance (actions to maintain physiological stability), monitoring (efforts to detect signs and symptoms), and management (actions to address functional health behaviors) [14].

The SCSE framework can help researchers and clinicians effectively address OAC-specific challenges related to self-care behaviors in patients undergoing OAC treatment. This approach facilitates the creation of a theory-grounded SE scale that can discern and evaluate complex situations in the context of OAC. These situations involve the behaviors patients need to adhere to maintain physical and mental stability over time (maintenance), monitor signs and symptoms, and implement appropriate actions for symptom management (management) [11,12]. Consequently, the SE within this framework will assess a patient’s confidence in performing these demanding self-care tasks associated with OAC, encompassing decision-making processes, cognitive functioning, and overall health literacy [11,12].

For example, some behaviors investigated within the 3 domains of self-care may reflect how well patients respond to tailored education and preparedness. Other behaviors might address shared challenges in maintaining a healthy status, monitoring changes in the body and mental well-being while on OAC treatment, and making informed decisions in response to unexpected changes. As the project expands, these behaviors could be further tested and integrated into OAC-specific self-care measures. Therefore, developing the Self-Care Self-Efficacy Index in Oral Anticoagulation Therapy Management (SCSE-OAC) aims to offer a comprehensive and nuanced understanding of patients’ SE in managing their OAC treatment effectively by using and incorporating the SCSE framework.

### Aims

This study aims to address the current gap in measuring SE in OAC treatment for NVAF. Existing measurement tools need more specificity and sensitivity to the unique challenges of OAC treatment, as recently highlighted [10]. To address this gap, the primary aim of this research protocol is to outline the design and methodology required to develop the SCSE-OAC in adults with NVAF. The study will provide cross-cultural validity and reliability of the developed measures for both English and Italian-speaking populations.

The secondary aim of this research protocol is to guide analyses to comprehensively describe patients’ SCSE levels in managing OAC for each native-speaking group participating in the study. The data collection rounds designed to validate the SCSE-OAC (primary aim) will lay the groundwork for future investigations into potential associations between patients’ SCSE and their sociodemographic or clinical variables. These findings will be instrumental in tailoring and implementing effective patient-centered educational interventions, ultimately enhancing adherence to OAC treatment and optimizing health outcomes.

## Methods

### Study Design

Following the STROBE (Strengthening the Reporting of Observational studies in Epidemiology) statement, a multiphase, mixed methods observational, and cross-sectional study design will be used. Additionally, the COSMIN (Consensus-based Standards for selecting health status Measurement Instruments) guidelines will be used to evaluate the measurement properties of SCSE-OAC [15].

**Figure 1:**
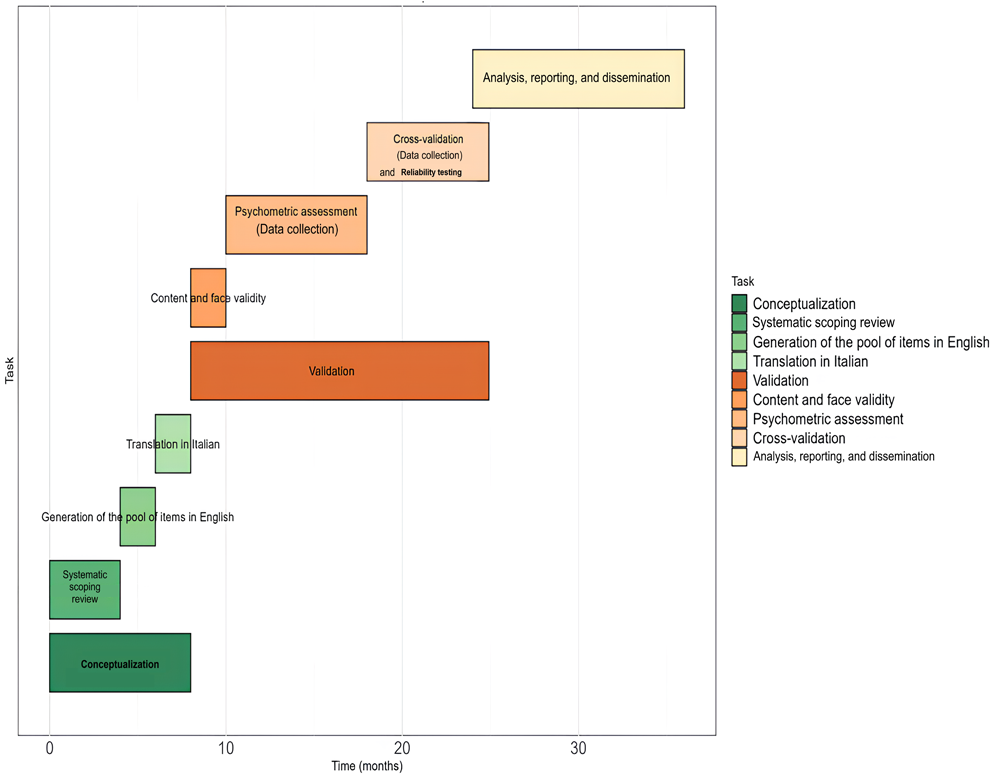
Gantt chart of the research activities related to the development and validation of the scale.

As depicted in Figure 1, the study will be structured into three main phases (1) conceptualization phase (phase 1), (2) validation phase (phase 2), and (3) reliability phase (phase 3). Phase 1 aims to establish a preliminary item’s pool for the SCSE-OAC and ensure cross-cultural validity for each native-speaking country involved in the research, encompassing both Italian and English versions. The focus of phase 2 is to provide evidence of the internal validity of the SCSE-OAC for each language group. In phase 3, the stability and consistency of the SCSE-OAC over time will be assessed.

### Phase 1: Conceptualization

In this initial phase (conceptualization), the primary objective is to generate a comprehensive list of behaviors that patients should perform in their daily self-care management of OAC. Specifically, we aim to develop a measure of patients’ SCSE, which pertains to their confidence in successfully executing appropriate self-care behaviors to address the unique challenges associated with chronic OAC treatment [16,17]. A total of 3 key substeps will be undertaken to achieve the primary aim of this study, using a mixed methods approach that integrates qualitative and quantitative methodologies to create the first version of the SCSE-OAC.

The first substep entails a systematic scoping literature review, which will help identify existing challenges and issues related to OAC self-care management in adults with NVAF as reported in previous researches. In the second substep, a group of patients with OAC treatment experience will be engaged to validate and potentially modify the initial set of behaviors identified in the literature review [18]. Similarly, in the third substep, input from health care experts with expertise in OAC management will be sought to further validate and refine the list of challenges or issues. Subsequently, the provisional version of the SCSE-OAC will be subject to cross-translation and cultural adaptation to ensure its relevance and applicability in the validation phase (phase 2) of the study.

More precisely, the systematic scoping literature review will be performed through the main academic research databases (PubMed, CINHAL, Scopus, and Web of Science) to identify the key pool of patients’ challenging behaviors in OAC management. In this stage, the “Population, Concept, Context” (PCC) framework will be used to develop the queries for each database [19,20] and be consistent with the framework to conduct scoping reviews [21].

In the second substep of this phase, purposive sampling of adult outpatients (18 years of age or older) with NVAF treated in OAC for at least 3 months will be involved in a small focus-group discussion to share the most relevant challenges they lived or perceived in the self-care management of OAC. In the methodology for our focus group discussions, we have chosen to start with a base number of 5 participants. This decision is grounded in qualitative research practices where smaller groups could facilitate more in-depth discussions, allowing each participant ample opportunity to share their experiences and insights [22]. This size is considered sufficient to begin exploring the range of patient perspectives on managing OAC, particularly given the specialized nature of the topic and the targeted patient population [22]. However, recognizing the importance of achieving comprehensive data saturation, our approach is designed to be flexible and iterative. After the initial focus group with 5 participants, we will continuously assess the information gathered for new and recurring themes. If additional perspectives are needed to achieve saturation—where no new information is observed in the data—we will conduct subsequent rounds of focus groups, incorporating more participants as required. This iterative process ensures that our research captures a full spectrum of patient experiences and challenges related to self-care management of OAC, thus enhancing the validity and applicability of our findings [23]. Therefore, a group of voluntary patients will be identified for each country involved in the study (Italy and Australia). A bilingual-trained moderator will be identified to guide the discussion for each patient group. Finally, a list of OAC challenges by patients’ views will be integrated with the literature review results, and the advice from the interviewed patients will contribute to defining the items’ wording. This phase is anticipated to end in October 2024.

After the patients’ focus-group discussion, a panel of experts (ie, physician, nurse, and pharmacist) will assess the relevance of each OAC-specific required behavior (item) identified in the previous phases. A multidisciplinary group of bilingual health care professionals with a minimum of 1 year of clinical experience in OAC management will be asked to rate the relevance of each item (ie, OAC challenges) on a 4-point Likert scale (1: completely disagree and 4: completely agree). Therefore, an overall interrater agreement among experts will be assessed through the Fleiss kappa index, and a value of more than 0.40 might be acceptable in a sample size ranging from 6 to 50 experts. Any divergence will be solved with consensus discussion.

In the last substep of phase 1, the provisional English version of the SCSE-OAC will be in parallel translated and cross-culturally adapted for each native-speaking country involved in the study (ie, Italian). Therefore, an independent group of bilingual experts will be involved in an iterative forward-backward translation process to ensure that the final version of the scale will be conceptually and linguistically correct and understandable to people of all levels of education by being culturally acceptable. Brislin’s translation procedure will be followed [24]. More specifically, 2 expert bilinguals who are native speakers of the target language will translate the English version of the SCSE-OAC into the Italian language, and a third expert will review the 2 translations to achieve the best possible reconciled translation. After that, another 2 bilingual experts who are native speakers of the target language with fluency in English will be involved in the back-translation of the previously reconciled translation to the original language (ie, Italian back to the English language). The results of all 3 tasks (translation, reconciliation, and back-translation) should be forwarded to a permanent panel of experts or translation coordinator to review the process. As a result of the translation process, 2 translated versions of SCSE-OAC will be available (1) SCSE-OAC for the Italian-speaking patients’ group and (2) SCSE-OAC for English-speaking patients’ group. At the end of this translation process, a pilot test will be performed to assess the readability and comprehensibility of the items for each native-speaking group (10-15 patients). Therefore, each patient group will be asked to fulfill the preliminary translated version of SCSE-OAC and highlight any items that are not clear to them. All comments and suggestions will be taken into account. This phase is anticipated to end in December 2024.

### Phase 2: Validation

This second phase (ie, validation phase) aims to define the construct intended to be measured, providing evidence of psychometrics validity for each translated version of SCSE-OAC. Two main substeps will be performed (1) content and face validity and (2) construct validity.

According to the COSMIN guideline, the content validity of the scale will be assessed based on the key criteria of relevance, comprehensiveness, and comprehensibility. In other words, each item will be analyzed by patients and health care professionals for content, meaning, wording, and format. As per the relevance criteria, we will ask to judge the items’ relevance for the construct of interest (ie, SCSE) on a 4-point Likert scale (1=not relevant, 2=somewhat relevant, 3=quite relevant, and 4=highly relevant), with the following question

Please evaluate the relevance of the following self-care behaviors in managing your oral anticoagulation medication. Which behaviors do you find relevant to your daily life with OAC treatment?

Content validity index (CVI) will be computed at the item and overall scale (ie, I-CVIs and S-CVI), considering a critical threshold value equal to 0.70 for I-CVIs and 0.80 for S-CVI. For each native-speaking country, a minimum of 15 patients will be enrolled to participate in the study.

The patients’ eligibility criteria will be adult outpatients (18 years of age or older) with ongoing treatment with OAC at the time of the investigation, with a primary clinical indication for long-term OAC for NVAF, treated for at least 3 months, and who are willing to participate in the study. Instead, patients with cognitive decline (ie, Six Item Screener Test with lower than 4 points) will be excluded. Likewise, a convenience sample of 10 voluntary professionals from all relevant disciplines (ie, physician, nurse, and pharmacist) with documented clinical experiences in OAC for at least 1 year will be involved. Sociodemographic and professional variables will be considered to provide a sample description. Finally, an open-ended question will be included to assess the comprehensive and comprehensibility criteria. More specifically, participants will be asked to evaluate whether the items together comprehensively cover the construct of interest and if the population of interest clearly understands the items and response options to evaluate the face validity. The responses will be systematically analyzed, coded, and compared through a qualitative content analysis approach. Data collection will be done through an e-survey.

Following the achievement of content and face validity, 3 forms of validity will be tested—structural, cross-cultural, and criterion validity. As per the structural validity, we will assess the dimensionality of the construct to be measured (ie, SCSE) by factor analysis. However, no clear hypotheses about the underlying dimensions of the construct in the OAC population are available. Therefore, a 2-fold cross-validation will be performed for each language group, using explorative factor analysis (EFA) to explore relationships between observed variables and underlying factors and identify the most plausible psychometric structure and confirmatory factor analysis (CFA) to confirm the most plausible structure derived from EFA. More specifically, the overall sample for each language group will be randomly resampled to generate a subsample A for performing EFA (roughly 50% of the original sample) and subsample B for performing a CFA using the most plausible psychometric structure derived from EFA (using the second 50% of the original sample).

### Sample Size for Phase 2

In the field of factor analysis, the sample size estimation for allowing researchers to perform factor analysis properly is based on a rule of thumb supported by the Monte Carlo simulation study of enrolling 10 patients per item and per language group [25]. As a result, the final required sample size will be based on the version of the SCSE-OAM derived from the content validity phase because it depends on the number of items encompassed in the scale. Considering previous similar research in patients with heart failure, 10 items were developed for measuring SCSE for adults with heart failure. We hypothesized a similar number of items. In this case, 100 patients per language group will be necessary for EFA and subsequently for CFA.

### Study Procedures for Cross-Sectional Data Collections

The 2 data collection rounds required for achieving construct validity will be performed using an e-survey or paper form based on patients’ preferences. E-survey is a more accessible and practical approach to gathering data from the desired group using a web survey link. The paper form will be delivered to patients in OAC who will prefer this approach. Considering that some patients could be older, the paper form could overcome the possible challenges that they could have in using an e-survey approach. A preidentified health care professional will oversee patients’ enrollment, get informed consent, and perform data management and monitoring. Each obtained paper form or electronically collected data will be registered (or transferred in the case of data collected via SurveyMonkey [SurveyMonkey Inc]) in REDCap (Research Electronic Data Capture) software (Vanderbilt University; ie, electronic case-report forms). REDCap works as a web-based repository of the data collected. Both SurveyMonkey and REDCap are compliant with the European General Data Protection Regulation.

### Eligibility Criteria for Phase 2

Inclusion criteria for participation in the study are (1) the patients must be aged 18 years or older, (2) have a confirmed diagnosis of NVAF, (3) be outpatients, and (4) have been receiving any OAC for at least 3 months before enrollment. Additionally, patients must provide informed consent. Exclusion criteria are those who have been treated with OAC for a brief period and are not on treatment anymore (ie, less than 3 months), patients with serious comorbidities (ie, Charlson Comorbidity Index, CCI >4), patients who have suspended OAC for surgery in the last 3 months, and those with cognitive impairment (assessed using a 6-item screener <4). Furthermore, a core set of sociodemographic and clinical variables will be collected to comprehensively describe the study sample.

### Measurements for Phase 2

The study will collect data on sociodemographic and clinical variables to comprehensively understand the participants. Sociodemographic variables will include age, sex (male or female), marital status, educational level, occupation status (employed or unemployed), socioeconomic status (income exemption—yes or no), and the country of the participants. Regarding clinical variables, the study will record the duration of OAC treatment, the type of OAC used (direct and indirect OAC), and the clinical condition that led to the prescription of OAC. For patients treated with vitamin K antagonists, the study will assess the time in therapeutic range (%) over the last 3 months to determine the effectiveness of treatment. Additionally, the occurrence of thromboembolic or hemorrhagic complications during OAC treatment within the last 3 months will be documented for both direct and indirect OAC users. In addition to the sociodemographic and clinical variables previously outlined, our study will also collect data on the number and types of other medications that participants are taking concurrently with their OAC. This inclusion acknowledges polypharmacy as a critical factor in the management and outcomes of patients with NVAF.

A total of 2 scoring systems, the CHA_2_DS_2_-VASc score [26] and the HAS-BLED (Hypertension, Abnormal liver/renal function, Stroke, Bleeding history or predisposition, Labile international normalized ratio, Elderly, Drugs/alcohol concomitantly) bleeding score [27] will be used to assess the risk factors associated with stroke and bleeding, respectively. The CHA_2_DS_2_-VASc score will help identify patients with a higher risk of stroke, where scores greater than 5 indicate a risk higher than 6.7%. On the other hand, the HAS-BLED bleeding score will be used to assess the risk of bleeding, with scores equal to or higher than 3 indicating a high risk of bleeding. By capturing these variables, the study aims to provide a comprehensive and detailed description of the participants in the research, enabling a thorough analysis of SCSE in OAC management.

### Data Analysis

The relevant number of factors to be retained in EFA data analysis will be done on the parameters of scree-test, parallel analysis, and factor rotation. More specifically, in the parallel analysis, if the eigenvalue of the original data’s factor is greater than the average of the eigenvalues of the parallel factor, that factor is retained. Likewise, the item retention will be assessed using factor loadings evaluations, where items with loadings >.40 will be retained. The structural model derived from EFA has to be used for both language groups in the CFA data analysis. The goodness of each model fit will be assessed based on the indexes of root-mean-square error of approximation (RMSEA; <.01) “excellent fit;” (<.05) “good fit;” (<.08) “mediocre fit;” comparative fit index (CFI) and Tucker-Lewis Index (TLI; >.90) and (>.95) [17,18]; SRMR (<.08) [19]; and chi-square test [25]. Furthermore, the multivariate normality of each model will be checked with the Mardia test, supposing that the multivariate skewness and kurtosis are significant. In the case of normality violation, a robust maximum likelihood estimator will be used.

After estimating the baseline model of scale for each language group, we will perform the Multi-Group Confirmatory Factor Analysis (MGCFA) to assess the cross-cultural validity. In other words, we will assess if the configural, metric, and scalar structure of SCSE-OAM is equivalent across 2 different language groups (English vs the Italian language). Achieving multi-group equivalence is pivotal to generating meaningful cross-group comparisons. Testing the measurement invariance means following a series of iterative analytical steps that bring from the largest model (where each language group has its parameters) to the more restricted one (where all the parameters are equal across the language group). This process stops when the model comparison is significant (ie, the large model fits the data significantly better than the more restricted one). A chi-square difference test will be performed to compare each model with the previous one to check whether a stronger invariance assumption holds. Data analysis will be performed in MPlus (Muthén & Muthén; version 8.1).

Clinical variables (ie, time in therapeutic range and absence of hemorrhagic and thromboembolic complications) will be used to estimate the cutoff points of adequate versus inadequate patients’ SCSE and assess criterion validity, which is the degree to which the score of the SCSE-OAM is an adequate reflection of good anticoagulation control. Sensitivity and specificity analyses will be performed under the receiver operator characteristic curve by using time in the therapeutic range and absence of hemorrhagic and thromboembolic complications as dichotomic outcomes (if the distribution of the outcomes will allow researchers to perform this analysis).

### Phase 3

The third phase of this research protocol focuses on evaluating the reliability and internal consistency of the SCSE-OAM over time. This phase, which is anticipated to end by December 2025, aims to ensure the stability of the developed scale. Two main criteria will be used for this assessment—internal consistency and reliability.

For internal consistency, the degree of interrelatedness among the items of the SCSE-OAM will be evaluated using Cronbach α. This index will be calculated for each unidimensional subscale, as well as for the overall scale. A Cronbach α value equal to or greater than 0.70 will be considered acceptable, indicating a strong internal consistency.

In assessing reliability, a test-retest study design will be used to determine the stability of the SCSE-OAM over time. After a 2-week interval, the second group of patients randomly selected from the previous one (sample B for CFA) will be asked to resubmit the SCSE-OAM. The minimum sample size for this test-retest reliability study will be set at 16 patients, with a power of 80%, a minimally acceptable reliability of 0.60, an expected reliability of 0.90, and α level of 0.05, considering a potential dropout rate of 10%. In addition to the scale responses, relevant clinical variables, such as the number of complications and any changes in the treatment regimen, will be collected to assess patients’ stability over time. The intraclass correlation coefficients with a 95% CI will be calculated using a 2-way random-effects model to evaluate the scale’s stability. This model enables us to assess the consistency of the SCSE-OAM when rated by different raters with characteristics similar to the selected raters in this study. By conducting this reliability phase, the research aims to validate the SCSE-OAM as a reliable and consistent tool for measuring SCSE in the context of OAC management.

### Ethical Considerations

This research protocol has obtained ethics approval from the Ethical Committee of Ospedale San Raffaele (164/INT/2022) and will be conducted in accordance with the principles outlined in the Helsinki Declaration, Good Clinical Practice, and European law on the right to privacy (GDPR 679/2016). The confidentiality of all collected data will be strictly ensured during both data collection and analysis, with information analyzed in an aggregate manner to protect participants’ identities. Ethics approval will be obtained from each participating institute, with Istituto di Ricovero e Cura a Carattere Scientifico (IRCCS) Policlinico San Donato acting as the owner of the data.

Informed consent will be a mandatory requirement for every participant enrolled in the study, and health care professionals will be responsible for providing clear and detailed information about the study’s objectives to the participants. Participants will have the right to withdraw their informed consent at any point during the study without facing any repercussions or penalties. This research prioritizes the protection of participants’ rights, privacy, and well-being, adhering to ethical guidelines and ensuring that the study is conducted with the utmost integrity and respect for all individuals involved.

## Results

The forthcoming validation of the SCSE-OAC is poised to significantly advance our understanding of SE in managing OAC among patients with NVAF. Anticipated results are expected to affirm the scale’s robust validity features, including its construct, criterion, and content validity, affirming its reliability across diverse patient demographics. Through exploratory and confirmatory factor analyses, the SCSE-OAC is projected to exhibit a coherent factor structure that aligns with theoretical expectations, reinforcing its use as a psychometrically sound tool for assessing SCSE in OAC.

Subsequent analyses are anticipated to unveil significant correlations between SCSE scores derived from the SCSE-OAC and key clinical outcomes, such as medication adherence rates, incidence of bleeding or thromboembolic events, and overall patient quality of life. These findings will underscore the critical role of SE in the self-management of OAC, suggesting that higher SE levels are associated with improved health outcomes and adherence behaviors. Moreover, the study is expected to elucidate the relationship between SE scores and sociodemographic characteristics, including age, education, and health literacy, providing valuable insights into how these factors may influence patients’ confidence and capabilities in managing their treatment regimens.

The significance of this study extends beyond the validation of a novel measurement tool. It promises to contribute substantially to the field of patient-centered care in anticoagulation management by offering health care professionals a reliable means to assess and enhance SE among their patients [1]. The SCSE-OAC will enable targeted educational and supportive interventions designed to bolster self-management skills by identifying, for instance, patients at risk of poor adherence due to low SE; therefore, mitigating risks associated with suboptimal treatment adherence. Furthermore, the scale’s validation across different languages and cultural contexts is anticipated to facilitate its global applicability, paving the way for its adoption in varied clinical settings and among diverse patient populations.

## Discussion

### Principal Findings

This research protocol addresses a critical gap in OAC management by focusing on developing and validating SCSE for OAC treatment (SCSE-OAC). OAC is an internationally recommended treatment for patients with NVAF and plays a crucial role in preventing venous thromboembolism and stroke while requiring the adjustment of lifestyle to the chronic treatment that requires adherence [2,9,28]. However, low levels of adherence and persistence to OAC have been observed by previous research [29], leading to potentially preventable thromboembolic and hemorrhagic complications [30]. Understanding the determinants underlying patients’ adherence to OAC, particularly their SE, is of paramount importance to designing personalized patient-centered educational interventions. The SCSE-OAC, being a specific and theory-grounded tool, will aid in capturing patients’ confidence in performing self-care behaviors and provide insights into the challenges they face in OAC management. Consequently, the study’s findings will contribute significantly to enhancing treatment adherence, safety, and overall health outcomes for patients with NVAF.

The study aims to provide a comprehensive SCSE-OAC for English- and Italian-speaking populations through a multiphase and mixed methods research design. In the conceptualization phase, a systematic literature review will help identify challenges and issues in OAC self-care management. Subsequently, qualitative and quantitative methodologies involving patients and experts will refine the initial items’ pool. The validation phase will focus on establishing the internal validity of the SCSE-OAC using Cronbach α, ensuring the tool’s reliability and consistency. Additionally, a test-retest reliability study will assess the stability of the SCSE-OAC over time. Overall, the study is expected to yield a robust and culturally adapted SCSE-OAC that can be applied to evaluate patients’ SE levels in OAC management for each native-speaking group involved in the research.

While previous studies have examined determinants of OAC adherence [29], the SCSE-OAC represents a novel contribution to the field. The SCSE-OAC could provide a more focused and contextually relevant assessment of patients’ self-care behaviors in OAC management because it will adopt a specific theoretical framework and develop a theory-grounded measure of SE. Existing tools used to measure SE have been diverse and general, lacking specificity for the challenges specific to OAC treatment [10]. The SCSE-OAC’s development and validation will enhance the field’s understanding of SE’s role in OAC management and could contribute to the implementation of tailored patient-centered educational interventions, ultimately improving treatment outcomes.

### Limitations

Several limitations may be encountered during the course of this research. First, despite the efforts made to include a diverse sample of patients, there might be some variations in the characteristics of participants from different geographic regions, potentially impacting the generalizability of the findings. Additionally, self-reported data may be subject to recall bias or social desirability bias, potentially affecting the accuracy of the responses. Moreover, the reliance on patients’ self-assessment for some clinical variables, such as complications during OAC treatment, might introduce some subjectivity to the data. We also have to acknowledge that the study’s cross-sectional design limits the ability to establish causality between patients’ SE and specific health outcomes.

An additional limitation to note is the geographical scope of our study, which initially includes participants from Italy and Australia. The choice to focus on these 2 countries was primarily driven by logistical and feasibility considerations, including the availability of research networks and resources to support the study’s implementation. While this focus provides valuable insights into the SCSE of patients managing OAC in these regions, it may not fully capture English speakers’ linguistic and cultural nuances in Europe, such as those in Ireland or the United Kingdom. We acknowledge that the distinct differences in Australian English compared to UK English, along with varying health care systems and patient education practices, could influence the generalizability of our findings to other English-speaking populations. Therefore, we will extend the validation phase of our project to include researchers and participants from other countries who wish to contribute to this body of knowledge. Interested parties are encouraged to contact the authors for potential collaboration, allowing authors to evaluate and integrate scaling-ups of the project accordingly. This adaptive approach aims to enhance the SCSE-OAC’s applicability and relevance across broader linguistic and cultural contexts, addressing an acknowledged limitation of this study design.

### Conclusions

This research protocol describes the development and validation of the SCSE-OAC in adults with NVAF by using a multiphase and mixed methods approach, guided by the SCSE framework, to ensure cross-cultural validity and reliability for both English and Italian-speaking populations. The SCSE-OAC will address the crucial need to assess patients’ confidence in managing their OAC treatment, enabling tailored patient-centered educational interventions and ultimately improving treatment adherence and health outcomes. These interventions are anticipated to markedly enhance treatment adherence and health outcomes, representing a significant stride in OAC management. The study will introduce a novel perspective in identifying and addressing individual patient needs, thus fostering better adherence and improving health outcomes, highlighting the importance of SCSE. The forthcoming efforts to validate the SCSE-OAC across diverse cultural and linguistic landscapes will amplify its applicability in global clinical and research settings and crucially contribute to establishing a universal standard for assessing SCSE in OAC management. This endeavor is pivotal for empowering health care professionals and researchers globally to devise efficacious and culturally attuned interventions.

## Abbreviations

CCI: Charlson Comorbidity Index
CDSES: Chronic Disease Self-Efficacy
CFA: confirmatory factor analysis
CFI: comparative fit index
COSMIN: Consensus-based Standards for selecting health status Measurement Instruments
CVI: content validity index
EFA: explorative factor analysis
GSE: General Self-Efficacy
HAS-BLED: Hypertension, Abnormal liver/renal function, Stroke, Bleeding history or predisposition, Labile international normalized ratio, Elderly, Drugs/alcohol concomitantly
IRCCS: Istituto di Ricovero e Cura a Carattere Scientifico
MGCFA: Multi-Group Confirmatory Factor Analysis
NVAF: nonvalvular atrial fibrillation
OAC: oral anticoagulation therapy
PCC: Population, Concept, Context
REDCap: Research Electronic Data Capture
RMSEA: root-mean-square error of approximation
SCSE: self-care self-efficacy
SCSE-OAC: Self-Care Self-Efficacy Index in Oral Anticoagulation Therapy Management
SE: self-efficacy
SEAMS: Self-Efficacy for Appropriate Medication Use Scale
STROBE: Strengthening the Reporting of Observational studies in Epidemiology
TLI: Tucker-Lewis Index

## Data Availability

The data sets generated and analyzed during this study will be available in the Zenodo repository once the study is completed.
